# Adverse Childhood Experiences and Health Conditions and Risk Behaviors Among High School Students — Youth Risk Behavior Survey, United States, 2023

**DOI:** 10.15585/mmwr.su7304a5

**Published:** 2024-10-10

**Authors:** Elizabeth A. Swedo, Sanjana Pampati, Kayla N. Anderson, Evelyn Thorne, Izraelle I. McKinnon, Nancy D Brener, Joi Stinson, Jonetta J. Mpofu, Phyllis Holditch Niolon

**Affiliations:** ^1^Division of Violence Prevention, National Center for Injury Prevention and Control, CDC; ^2^Division of Adolescent and School Health, National Center for Chronic Disease Prevention and Health Promotion, CDC; ^3^Office of Strategy and Innovation, National Center for Injury Prevention and Control, CDC; ^4^Epidemic Intelligence Service, CDC

## Abstract

Adverse childhood experiences (ACEs) are preventable, potentially traumatic events occurring before age 18 years. Data on ACEs among adolescents in the United States have primarily been collected through parent report and have not included important violence-related ACEs, including physical, sexual, and emotional abuse. This report presents the first national prevalence of self-reported ACEs among U.S. high school students aged <18 years, estimates associations between ACEs and 16 health conditions and risk behaviors, and calculates population-attributable fractions of ACEs with these conditions and behaviors using cross-sectional, nationally representative 2023 Youth Risk Behavior Survey data. Exposures were lifetime prevalence of individual (emotional, physical, and sexual abuse; physical neglect; witnessed intimate partner violence; household substance use; household poor mental health; and incarcerated or detained parent or guardian) ACEs and cumulative ACEs count (zero, one, two or three, or four or more). Health conditions and risk behaviors included violence risk factors, substance use, sexual behaviors, weight and weight perceptions, mental health, and suicidal thoughts and behaviors. Bivariate analyses assessed associations between individual and cumulative ACEs and demographics. Adjusted prevalence ratios assessed associations between cumulative ACEs and health conditions and risk behaviors, accounting for demographics. Population-attributable fractions were calculated to determine the potential reduction in health conditions and risk behaviors associated with preventing ACEs. ACEs were common, with approximately three in four students (76.1%) experiencing one or more ACEs and approximately one in five students (18.5%) experiencing four or more ACEs. The most common ACEs were emotional abuse (61.5%), physical abuse (31.8%), and household poor mental health (28.4%). Students who identified as female; American Indian or Alaska Native; multiracial; or gay or lesbian, bisexual, questioning, or who describe their sexual identity in some other way experienced the highest number of ACEs. Population-attributable fractions associated with experiencing ACEs were highest for suicide attempts (89.4%), seriously considering attempting suicide (85.4%), and prescription opioid misuse (84.3%). ACEs are prevalent among students and contribute substantially to numerous health conditions and risk behaviors in adolescence. Policymakers and public health professionals can use these findings to understand the potential public health impact of ACEs prevention to reduce adolescent suicidal behaviors, substance use, sexual risk behaviors, and other negative health conditions and risk behaviors and to understand current effects of ACEs among U.S. high school students.

## Introduction

Adverse childhood experiences (ACEs) are preventable, potentially traumatic events occurring before age 18 years (*1*). For nearly three decades, considerable research, particularly among adults, has demonstrated the importance of ACEs’ contributions to negative health outcomes and diminished life opportunities (*2*,*3*). Retrospective surveys of adults have established that ACEs are prevalent: Approximately two thirds of U.S. adults have experienced at least one ACE and 17% of U.S. adults have experienced four or more ACEs (*4*). Despite the volume of research supporting strong associations between ACEs and negative outcomes in adulthood, relatively few studies have examined the role of ACEs in child and adolescent health (*5*). ACEs are associated with increased likelihood of numerous risk factors and health outcomes, including being in physical fights and carrying weapons (*6*); smoking, alcohol use, and illicit drug use in both adolescence and adulthood (*2*); early sexual initiation, teenage pregnancy, sexually transmitted infections, and multiple sexual partners (*2*); overweight or obesity (*2*); and various mental health conditions or symptoms, as well as suicide risk, across the lifespan (*5*,*7*).

Population-level estimates of ACEs among adolescents and their effects have largely been limited to parent-report or a small number of ACEs (*8*). Collecting and analyzing self-reported ACEs data among adolescents is valuable for several reasons, most notably the proximity of the event to the time of report and adolescents’ knowledge of their own experiences that might be unknown to or not disclosed by their parents (*9*). Retrospective reports of ACEs on surveys, such as the Behavioral Risk Factor Surveillance System (BRFSS), require adults to recall events that occurred decades ago, introducing the potential for significant recall bias (*9*). Collection of ACE data among adolescents decreases recall bias, improves the ability to track trends in ACEs over time, and permits quicker assessment of the impact of current prevention and mitigation efforts in disproportionately affected populations (*9*). Collection of self-reported ACEs data among adolescents, and not parent-proxy reporters, might improve accuracy of estimates: Adolescent-reported data potentially captures abuse- or neglect-related experiences that might have been perpetrated by the proxy reporters, and sensitive experiences not disclosed by adolescents to their parent.

This report presents the first lifetime national prevalence of self-reported individual and cumulative ACEs among U.S. high school students aged <18 years, associations between cumulative ACE exposure and negative health conditions and risk behaviors in adolescence, and population-attributable fractions related to ACEs for each condition and behavior. Policymakers and public health professionals can use this information to understand current prevalence of ACEs among U.S. high school students, and the proportion of negative health conditions and risk behaviors that could potentially be reduced or eliminated by implementing evidence-based strategies and approaches to prevent ACEs and mitigate their consequences.

## Methods

### Data Source

This report includes data from the 2023 Youth Risk Behavior Survey (YRBS) (N = 20,103), a cross-sectional, school-based survey conducted biennially since 1991. Each survey year, CDC collects data from a nationally representative sample of public and private school students in grades 9–12 in the 50 U.S. states and the District of Columbia. Additional information about YRBS sampling, data collection, response rates, and processing is available in the overview report of this supplement (*8*). Prevalence estimates for ACEs for the overall study population and by sex, race and ethnicity, grade, and sexual identity are available at https://nccd.cdc.gov/youthonline/App/Default.aspx. The full YRBS questionnaire, datasets, and documentation are available at https://www.cdc.gov/yrbs/index.html. Institutional review boards at CDC and ICF, the survey contractor, approved the protocol for YRBS. Data collection was conducted consistent with applicable Federal law and CDC policy.*

### Measures

Information about question content and coding for all demographics, ACEs, and included health conditions and risk behaviors is presented (Table 1). Students self-reported lifetime experiences of eight ACEs (emotional, physical, and sexual abuse; physical neglect; witnessing intimate partner violence; household substance use; household poor mental health; and parent or guardian incarcerated or detained). Questions align with and were adapted from the original ACEs included in the seminal CDC-Kaiser Permanente ACEs Study (*3*) and subsequently used for adult retrospective data collection from the BRFSS (*4*). Slight adaptations were made to ACEs questions to align with age of respondent (i.e., changes to the question stem from “before you were 18 years of age” to “during your life”), and to reduce the number of questions used to capture sexual abuse and household substance use. ACEs questions were cognitively tested with high school students to ensure fidelity to question intention and suitability for adolescent populations; cognitive testing results are available (https://stacks.cdc.gov/view/cdc/150784). In addition to examination of the presence of individual ACEs, a cumulative ACEs count (cumulative ACEs) was calculated (zero, one, two or three, or four or more) following CDC guidelines for coding ACEs responses using YRBS data (*10*).

**TABLE 1:** Questions, response options, and analytic coding for health outcomes, risk behaviors, and adverse childhood experiences among high school students aged <18 years, by variable assessed — Youth Risk Behavior Survey, United States, 2023

Variable	Question	Response option	Analytic coding
**Adverse childhood experience**			
Emotional abuse	During your life, how often has a parent or other adult in your home insulted you or put you down?	Never, rarely, sometimes, most of the time, or always	Yes (rarely, sometimes, most of the time, or always) versus no (never)
Physical abuse	During your life, how often has a parent or other adult in your home hit, beat, kicked, or physically hurt you in any way?	Never, rarely, sometimes, most of the time, or always	Yes (rarely, sometimes, most of the time, or always) versus no (never)
Sexual abuse	Has an adult or person at least 5 years older than you ever forced you to do sexual things that you did not want to do? (Count such things as kissing, touching, or being made to have sexual intercourse.)	Yes or no	Yes versus no
Physical neglect	During your life, how often has there been an adult in your household who tried hard to make sure your basic needs were met, such as looking after your safety and making sure you had clean clothes and enough to eat?	Never, rarely, sometimes, most of the time, or always	Yes (never or rarely) versus no (sometimes, most of the time, and always)*
Witnessed intimate partner violence	During your life, how often have your parents or other adults in your home slapped, hit, kicked, punched, or beat each other up?	Never, rarely, sometimes, most of the time, or always	Yes (rarely, sometimes, most of the time, or always) versus no (never)
Household substance use	Have you ever lived with a parent or guardian who was having a problem with alcohol or drug use?	Yes or no	Yes versus no
Household poor mental health	Have you ever lived with a parent or guardian who had severe depression, anxiety, or another mental illness, or was suicidal?	Yes or no	Yes versus no
Parent or guardian incarcerated or detained	Have you ever been separated from a parent or guardian because they went to jail, prison, or a detention center?	Yes or no	Yes versus no
Cumulative ACEs count	Cumulative ACEs count = (emotional abuse + physical abuse + sexual abuse + physical neglect + witnessed IPV + household substance use + household poor mental health + incarcerated parent or guardian)	Total count possible is 8^†^	Categorization for ACEs: 0, 1, 2 or 3, or ≥4
**Health outcome or risk behavior**			
Carried a weapon at school during the past 30 days	During the past 30 days, on how many days did you carry a weapon such as a gun, knife, or club on school property?	0 days, 1 day, 2−3 days, 4−5 days, or ≥6 days	Yes (1 day, 2−3 days, 4−5 days, or ≥6 days) versus no (0 days)
Was in a physical fight during the past 12 months	During the past 12 months, how many times were you in a physical fight?	0 times, 1 time, 2−3 times, 4−5 times, 6−7 times, 8−9 times, 10− 11 times, or ≥12 times	Yes (1 time, 2−3 times, 4−5 times, 6−7 times, 8−9 times, 10−11 times, or ≥12 times) versus no (0 times)
Current electronic vapor product use during the past 30 days	During the past 30 days, on how many days did you use an electronic vapor product?	0 days, 1−2 days, 3−5 days, 6−9 days, 10−19 days, 20−29 days, or all 30 days	Yes (1−2 days, 3−5 days, 6−9 days, 10−19 days, 20−29 days, or all 30 days) versus no (0 days)
Current alcohol use during the past 30 days	During the past 30 days, on how many days did you have at least one drink of alcohol?	0 days, 1−2 days, 3−5 days, 6−9 days, 10−19 days, 20−29 days, or all 30 days	Yes (1−2 days, 3−5 days, 6−9 days, 10−19 days, 20−29 days, or all 30 days) versus no (0 days)
Current binge drinking during the past 30 days	During the past 30 days, on how many days did you have ≥4 drinks of alcohol in a row, that is, within a couple of hours (if you are female) or ≥5 drinks of alcohol in a row, that is, within a couple of hours (if you are male)?	0 days, 1 day, 2 days, 3−5 days, 6−9 days, 10−19 days, or ≥20 days	Yes (1 day, 2 days, 3−5 days, 6−9 days, 10−19 days, or ≥20 days) versus no (0 days)
Current prescription opioid misuse during the past 30 days	During the past 30 days, how many times did you take prescription pain medicine without a doctor’s prescription or differently than how a doctor told you to use it?	0 times, 1−2 times, 3−9 times, 10−19 times, 20−39 times, or ≥40 times	Yes (1−2 times, 3−9 times, 10−19 times, 20−39 times, or ≥40 times) versus no (0 times)
Alcohol or drug use before last sexual intercourse	Did you drink alcohol or use drugs before you had sexual intercourse the last time?	I have never had sexual intercourse, yes, or no	Yes versus no (no or I have never had sexual intercourse)
Currently sexually active with multiple people during the past 3 months	During the past 3 months, with how many people did you have sexual intercourse?	I have never had sexual intercourse, I have had sexual intercourse, but not during the past 3 months, 1 person, 2 persons, 3 persons, 4 persons, 5 persons, or ≥6 persons	Yes (2 people, 3 people, 4 people, 5 people, or 6 or more people) versus no (I have never had sexual intercourse, I have had sexual intercourse, but not during the past 3 months, or 1 person)
Did not use a condom during last sexual intercourse	The last time you had sexual intercourse, did you or your partner use a condom?	I have never had sexual intercourse, yes, or no	Yes (no [did not use condom]) versus no (yes [used a condom] or I have never had sexual intercourse)
Underweight	How tall are you without your shoes on? How much do you weigh without your shoes on?	Numeric entry of height in feet and inches; weight in pounds	BMI calculated <5th percentile using self-reported height and weight^§^
Overweight or obesity	How tall are you without your shoes on? How much do you weigh without your shoes on?	Numeric entry of height in feet and inches; weight in pounds	BMI calculated ≥85th percentile using self-reported height and weight^§^
Self-perceived to be underweight	How do you describe your weight?	Very underweight, slightly underweight, about the right weight, slightly overweight, or very overweight	Self-perceived underweight (very underweight or slightly underweight) versus self-perceived “about the right” weight
Self-perceived to be overweight	How do you describe your weight?	Very underweight, slightly underweight, about the right weight, slightly overweight, or very overweight	Self-perceived overweight (slightly overweight or very overweight) versus self-perceived “about the right” weight
Persistent feelings of sadness or hopelessness during the past 12 months	During the past 12 months, did you ever feel so sad or hopeless almost every day for 2 weeks or more in a row that you stopped doing some usual activities?	Yes or no	Yes versus no
Seriously considered attempting suicide during the past 12 months	During the past 12 months, did you ever seriously consider attempting suicide?	Yes or no	Yes versus no
Attempted suicide during the past 12 months	During the past 12 months, how many times did you actually attempt suicide?	0 times, 1 time, 2−3 times, 4−5 times, or ≥6 times	Yes (1 time, 2−3 times, 4−5 times, or ≥6 times) versus no (0 times)

**Abbreviations:** ACEs = adverse childhood experiences; BMI = body mass index.

* Physical neglect is reverse coded and includes both “never” and “rarely” to align with previously published measures of neglect. https://www.cdc.gov/violenceprevention/aces/ace-brfss.html

^†^ Cumulative ACE count calculated only for participants with complete data on at least five individual ACEs.

^§^ BMI for each student was calculated from self-reported height and weight and then an obesity indicator and an overweight indicator were categorized based on sex- and age-specific reference data from the 2000 CDC Extended BMI-for-Age Growth Charts. https://www.cdc.gov/growthcharts/clinical_charts.htm

Students also self-reported on 16 measures across a spectrum of risk behaviors and health conditions. These included carrying a weapon at school, being in a physical fight, multiple forms of substance use (i.e., current electronic vapor product use, current alcohol use, current binge drinking, and current prescription opioid misuse), sexual behaviors (i.e., alcohol or drug use before last sexual intercourse, currently sexually active with multiple partners, and did not use a condom during last sexual intercourse), weight and perceived weight status, persistent feelings of sadness or hopelessness, and suicide risk (i.e., seriously considered attempting suicide or attempted suicide). Most questions referenced conditions or behaviors that took place during the past 12 months or past 30 days, increasing the chances that the condition or behavior took place after initial ACE exposure.

Demographic variables included sex (female or male) and race and ethnicity (American Indian or Alaska Native [AI/AN], Asian, Black or African American [Black], White, Hispanic or Latino [Hispanic], and multiracial [selected >1 racial category]). (Persons of Hispanic or Latino origin might be of any race but are categorized as Hispanic; all racial groups are non-Hispanic.) Prevalence estimates for Native Hawaiian or other Pacific Islander students had denominators <30 and were therefore considered statistically unreliable and were suppressed (*8*). Other demographic variables included age (≤14, 15, 16, and 17 years), and sexual identity (heterosexual, gay or lesbian, bisexual, questioning [I am not sure about my sexual identity/questioning], or describe identity in some other way [I describe my identity some other way]).

### Analysis

The analytic sample was restricted to those aged <18 years (n = 17,838) to ensure the adversity occurred during childhood. Weighted prevalence and 95% CIs for individual and cumulative ACEs count, overall and by each demographic, are presented. Demographic differences in the prevalence of individual ACEs and cumulative ACEs count were examined using pairwise *t*-test analyses. All prevalence estimates and measures of association used Taylor series linearization. Cumulative ACEs counts were only calculated for participants with complete data on at least five individual ACEs.

The weighted prevalence and 95% CI of each health condition and risk behavior by cumulative ACEs count are presented. Adjusted prevalence ratios (aPRs) were calculated using logistic regression with predicted marginals; models fit cumulative ACEs count as the independent variable and each risk behavior or health condition as the dependent variable, adjusting for sex, race and ethnicity, age, and sexual identity. Population-attributable fractions, adjusted for aforementioned model covariates, were calculated using Miettinen’s formula, aPRs, and weighted prevalence estimates of each health condition and risk behavior by each cumulative ACEs count level (Supplementary Table, https://stacks.cdc.gov/view/cdc/160323). These population-attributable fractions were used to ascertain the percentage reduction in the number of observed cases of each outcome that would be expected if ACEs exposure were incrementally reduced or eliminated in the study population (*7*,*11*). Findings were considered statistically significant if p<0.05. Prevalence ratios were considered statistically significant if 95% CI did not cross the value of 1.0. All analyses except estimates of population-attributable fractions were conducted in SAS-callable SUDAAN (version 11.0.3; RTI International) using sample weights to account for complex survey design.

## Results

The most common ACEs were emotional abuse (61.5%), physical abuse (31.8%), and household poor mental health (28.4%) (Table 2). Experiences of specific ACEs varied by demographic characteristics. All ACEs, except physical neglect, were more common among female students compared with male students. Unique patterns were observed by race and ethnicity for individual ACEs. For example, AI/AN students had the highest prevalence of witnessed intimate partner violence (28.7%) and household substance use (34.2%) but one of the lowest prevalence estimates of physical abuse (35.7%). Asian students had the lowest prevalence of sexual abuse (4.3%), witnessed intimate partner violence (17.1%), household substance use (12.4%), household poor mental health (13.2%), and parent or guardian incarcerated or detained (4.6%), but had the highest prevalence of physical abuse (38.2%), along with Black students (38.2%). Prevalence differed by age for four out of eight ACEs; household substance use and household poor mental health were more commonly reported by students aged 17 years (26.4% and 30.3%, respectively) compared with students aged ≤14 years (21.3% and 24.7%, respectively). Students who identified as gay, lesbian, bisexual, or questioning (LGBQ+) had higher prevalence of all ACEs except physical neglect compared with heterosexual students. Heterosexual students (8.6%) experienced a higher prevalence of physical neglect than bisexual students (6.8%) and students who describe their identity in some other way (3.9%). Bisexual students experienced a higher prevalence of sexual abuse (19.6%) and household substance use (40.6%) compared with gay and lesbian students (10.0% and 30.1%, respectively). Students who describe their identity in some other way had higher prevalence of emotional abuse (85.0%), household substance use (43.0%), and household poor mental health (56.4%) compared with gay and lesbian students (77.0%, 30.1%, and 42.0%, respectively).

**TABLE 2:** Lifetime prevalence of individual types of adverse childhood experiences among high school students aged <18 years, by sociodemographic characteristics — Youth Risk Behavior Survey, United States, 2023*

Characteristic	Weighted % (95% CI)	Adverse childhood experience category weighted % (95% CI)							
		Emotional abuse % (95% CI)	Physical abuse % (95% CI)	Sexual abuse % (95% CI)	Physical neglect % (95% CI)	Witnessed IPV % (95% CI)	Household substance use % (95% CI)	Household poor mental health % (95% CI)	Parent or guardian incarcerated or detained % (95% CI)
**Sex**									
Female	48.5 (46.4–50.7)	69.1 (66.1–72.0)	34.1 (31.6–36.7)	11.8 (10.5–13.1)	8.4 (6.6–10.7)	23.1 (21.0–25.3)	29.7 (27.2–32.4)	35.5 (32.9–38.2)	15.9 (13.5–18.5)
Male	51.5 (49.3–53.6)	54.3 (51.3–57.3)^†^	29.7 (27.2–32.4)^†^	2.7 (2.0–3.7)^†^	10.1 (8.3–12.2)^†^	14.4 (12.7–16.4)^†^	20.7 (18.4–23.2)^†^	21.7 (19.5–24.2)^†^	13.2 (11.1–15.7)^†^
**Race and ethnicity^§^**									
American Indian or Alaska Native	0.4 (0.2–0.5)	56.5 (33.2–77.3)	35.7 (19.6–55.9)	7.2 (3.4–14.9)	8.0 (3.4–17.5)	28.7 (15.8–46.5)	34.2 (18.3–54.5)^¶^	29.9 (16.0–48.9)	17.4 (9.7–29.4)^¶^
Asian	4.3 (2.9–6.4)	66.0 (60.7–71.0)**^,††^	38.2 (32.6–44.2)^§§^	4.3 (2.4–7.8)^††^	7.4 (4.7–11.6)**^,††^	17.1 (13.7–21.1)^¶¶^	12.4 (8.4–18.0)^††,§§,¶¶^	13.2 (9.2–18.7)**^,††,§§,¶¶^	4.6 (2.2–9.4) **^,††,§§,¶¶^
Black or African American	13.4 (9.3–18.9)	55.6 (48.8–62.1)***^,†††^	38.2 (30.9–46.0)***	6.1 (4.6–7.9)^§§§^	12.0 (9.2–15.5)***^,†††^	18.6 (16.2–21.3)^†††^	16.1 (14.3–18.1)***^,†††,§§§^	19.1 (16.9–21.4)***^,†††,§§§^	17.0 (14.3–20.0)***
White	47.3 (40.6–54.1)	65.4 (62.3–68.4)	27.0 (24.9–29.1)	6.3 (4.9–8.1)	7.4 (5.7–9.6)	17.2 (15.0–19.5)	29.2 (26.0–32.6)	33.3 (29.9–36.9)	12.6 (10.3–15.3)
Hispanic or Latino	27.9 (23.2–33.3)	57.3 (53.9–60.7)^¶¶¶,^****	33.6 (30.6–36.7)^¶¶¶^	9.1 (8.1–10.2)^¶¶¶^	12.1 (9.6–15.2)^¶¶¶,^****	19.4 (17.0–21.9)****	24.8 (21.8–28.1)^¶¶¶^	27.0 (23.9–30.4)^¶¶¶,^****	16.8 (14.0–19.9)^¶¶¶^
Multiracial	6.3 (4.5–8.7)	67.0 (60.4–73.0)	37.5 (33.3–42.0)^††††^	8.2 (5.9–11.2)	6.0 (4.0–8.9)	25.4 (21.3–29.9)^††††^	28.2 (23.1–33.9)	37.1 (31.5–43.0)	20.1 (14.2–27.6)^††††^
**Age, yrs**									
≤14	14.3 (12.6–16.1)	62.7 (58.0–67.1)	31.9 (28.1–36.0)	7.4 (6.3–8.8)	10.0 (7.7–12.9)	17.4 (14.7–20.4)	21.3 (17.8–25.4)^§§§§,¶¶¶¶^	24.7 (21.5–28.1)^¶¶¶¶,^*****	12.9 (10.3–15.9)
15	28.7 (27.0–30.3)	59.1 (56.5–61.7)^†††††^	30.9 (28.4–33.6)	6.4 (5.2–7.8)	8.0 (6.5–9.9)^†††††^	18.4 (16.2–20.7)	24.7 (22.4–27.2)	29.0 (26.2–31.8)	14.1 (11.9–16.5)
16	29.4 (27.9–31.0)	61.0 (57.1–64.7)	33.3 (30.3–36.5)	7.0 (5.6–8.7)	9.5 (7.4–12.0)	18.2 (16.1–20.6)	26.1 (23.1–29.3)	27.8 (24.7–31.3)	15.2 (12.7–18.1)
17	27.6 (26.1–29.2)	63.9 (59.7–67.8)	31.2 (28.2–34.4)	7.6 (6.2–9.2)	10.2 (7.9–13.0)	19.9 (17.1–23.1)	26.4 (23.6–29.5)	30.3 (26.8–34.0)	15.1 (12.5–18.2)
**Sexual identity**									
Heterosexual	74.9 (72.6–77.1)	56.3 (53.8–58.8)^§§§§§,¶¶¶¶¶,^******^,††††††^	28.3 (26.3–30.4)^§§§§§,¶¶¶¶¶,^******^,††††††^	4.1 (3.5–4.7)^§§§§§,¶¶¶¶¶,^******^,††††††^	8.6 (7.7–9.6)^¶¶¶¶¶,^******	14.8 (13.8–16.0)^§§§§§,¶¶¶¶¶,^******^,††††††^	20.5 (18.7–22.5)^§§§§§,¶¶¶¶¶,^******^,††††††^	22.4 (20.6–24.3)^§§§§§,¶¶¶¶¶,^******^,††††††^	12.0 (10.6–13.6)^§§§§§,¶¶¶¶¶,^******^,††††††^
Gay or lesbian	4.1 (3.6–4.7)	77.0 (69.0–83.4)^§§§§§§^	40.3 (34.3–46.6)	10.0 (6.6–14.9^¶¶¶¶¶¶^	9.7 (6.1–14.9)^§§§§§§^	26.7 (22.7–31.1)*******	30.1 (24.2–36.8)^§§§§§§,¶¶¶¶¶¶^	42.0 (34.7–49.8)^§§§§§§^	17.6 (12.7–23.9)
Bisexual	11.8 (10.7–13.1)	81.5 (78.2–84.3)	46.0 (42.9–49.1)	19.6 (16.6–23.1)^†††††††^	6.8 (5.3–8.7)^§§§§§§§^	29.1 (25.1–33.4)^†††††††^	40.6 (36.3–45.0)^†††††††^	48.9 (45.1–52.8)	19.4 (15.4–24.2)
Questioning	4.7 (4.1–5.4)	78.8 (73.1–83.6)	43.9 (37.3–50.7)	12.6 (9.4–16.7)	7.0 (4.4–11.0)	20.5 (16.7–24.9)^¶¶¶¶¶¶¶^	31.7 (25.8–38.3)^¶¶¶¶¶¶¶^	42.5 (36.1–49.3)^¶¶¶¶¶¶¶^	16.7 (13.0–21.2)
Describe identity in some other way	4.5 (3.8–5.2)	85.0 (79.2–89.4)	48.0 (39.5–56.6)	17.7 (10.2–28.8)	3.9 (2.1–7.0)	31.2 (24.3–39.1)	43.0 (34.2–52.1)	56.4 (46.6–65.7)	20.6 (13.6–29.9)
**Total**	**—**	**61.5 (58.8–64.1)**	**31.8 (29.7–34.0)**	**7.1 (6.3–7.9)**	**9.3 (7.6–11.4)**	**18.6 (17.0–20.4)**	**25.1 (22.9–27.4)**	**28.4 (26.0–30.9)**	**14.5 (12.4–16.9)**

**Abbreviation:** IPV = intimate partner violence.

* N = 17,838 respondents aged <18 years. The total number of students answering each question varied. Data might be missing because 1) the question did not appear in that student’s questionnaire, 2) the student did not answer the question, or 3) the response was set to missing because of an out-of-range response or logical inconsistency. Percentages in each category are calculated on the known data.

^†^ Male students significantly differed from female students, based on *t*-test analysis with Taylor series linearization (p<0.05).

^§^ Persons of Hispanic or Latino origin might be of any race but are categorized as Hispanic; all racial groups are non-Hispanic.

^¶^ American Indian or Alaska Native students significantly differed from Asian students, based on *t*-test analysis with Taylor series linearization (p<0.05).

** Asian students significantly differed from Black or African American students, based on *t*-test analysis with Taylor series linearization (p<0.05).

^††^ Asian students significantly differed from Hispanic or Latino students, based on *t*-test analysis with Taylor series linearization (p<0.05).

^§§^ Asian students significantly differed from White students, based on *t*-test analysis with Taylor series linearization (p<0.05).

^¶¶^ Asian students significantly differed from multiracial students, based on *t*-test analysis with Taylor series linearization (p<0.05).

*** Black or African American students significantly differed from White students, based on *t*-test analysis with Taylor series linearization (p<0.05).

^†††^ Black or African American students significantly differed from multiracial students, based on *t*-test analysis with Taylor series linearization (p<0.05).

^§§§^ Black or African American students significantly differed from Hispanic or Latino students, based on *t*-test analysis with Taylor series linearization (p<0.05).

^¶¶¶^ Hispanic or Latino students significantly differed from White students (p<0.05).

**** Hispanic or Latino students significantly differed from multiracial students (p<0.05).

^††††^ Multiracial students significantly differed from White students (p<0.05).

^§§§§^ Students aged ≤14 years significantly differed from students aged 16 years, based on *t*-test analysis with Taylor series linearization (p<0.05).

^¶¶¶¶^ Students aged ≤14 years significantly differed from students aged 17 years, based on *t*-test analysis with Taylor series linearization (p<0.05).

***** Students aged ≤14 years significantly differed from students aged 15 years, based on *t*-test analysis with Taylor series linearization (p<0.05).

^†††††^ Students aged 15 years significantly differed from students aged 17 years, based on *t*-test analysis with Taylor series linearization (p<0.05).

^§§§§§^ Heterosexual students significantly differed from gay or lesbian students, based on *t*-test analysis with Taylor series linearization (p<0.05).

^¶¶¶¶¶^ Heterosexual students significantly differed from bisexual students, based on *t*-test analysis with Taylor series linearization (p<0.05).

****** Heterosexual students significantly differed from students who describe their identity some other way, based on *t*-test analysis with Taylor series linearization (p<0.05).

^††††††^ Heterosexual students significantly differed from students with who were questioning, based on *t*-test analysis with Taylor series linearization (p<0.05).

^§§§§§§^ Gay or lesbian students significantly differed from students who describe their identity some other way, based on *t*-test analysis with Taylor series linearization (p<0.05).

^¶¶¶¶¶¶^ Gay or lesbian students significantly differed from bisexual students, based on *t*-test analysis with Taylor series linearization (p<0.05).

******* Gay or lesbian students significantly differed from questioning students, based on *t*-test analysis with Taylor series linearization (p<0.05).

^†††††††^ Bisexual students significantly differed from questioning students, based on *t*-test analysis with Taylor series linearization (p<0.05).

^§§§§§§§^ Bisexual students significantly differed from students who describe their identity some other way, based on *t*-test analysis with Taylor series linearization (p<0.05).

^¶¶¶¶¶¶¶^ Students who describe their identity in some other way significantly differed from students who were questioning, based on *t*-test with Taylor series linearization (p<0.05).

Three in four students (76.1%) experienced at least one ACE and nearly one in five (18.5%) experienced four or more ACEs (Table 3). The prevalence of four or more ACEs was highest in female (23.9%), AI/AN (28.1%), multiracial (25.9%), and LGBQ+ students, particularly students who describe their identity in some other way (38.6%) and bisexual students (35.1%). AI/AN students had the highest prevalence of experiencing zero ACEs (30.9%).

**TABLE 3:** Cumulative adverse childhood experiences among high school students aged <18 years, by sociodemographic characteristics — Youth Risk Behavior Survey, United States, 2023

Characteristic	Cumulative adverse childhood experiences* weighted % (95% CI)			
	0 % (95% CI)	1 % (95% CI)	2 or 3 % (95% CI)	≥4 % (95% CI)
**Sex**				
Female	18.3 (16.4–20.3)	23.8 (21.9–25.7)	34.1 (31.6–36.6)	23.9 (21.7–26.2)
Male	29.3 (27.1–31.6)^†^	25.0 (23.3–26.7)	32.5 (30.1–34.9)	13.3 (11.3–15.6)^†^
**Race and ethnicity^§^**				
American Indian or Alaska Native	30.9 (10.8–62.3)	16.8 (7.8–32.5)	24.2 (12.4–41.9)	28.1 (14.8–46.9)^¶^
Asian	26.3 (21.6–31.6)**	24.8 (20.1–30.1)	37.7 (33.2–42.4)**	11.3 (7.9–15.8)**^,††,§§,¶¶^
Black or African American	25.6 (21.7–30.0)***	21.9 (18.7–25.5)^†††^	36.7 (32.0–41.8)^†††^	15.7 (13.2–18.6)***
White	23.3 (20.8–26.0)	25.7 (23.8–27.7)	32.0 (29.5–34.6)	19.0 (16.2–22.1)
Hispanic or Latino	24.5 (22.1–27.0)^§§§^	23.0 (21.1–25.1)^¶¶¶^	33.4 (31.0–35.8)	19.1 (16.5–22.0)^§§§^
Multiracial	16.7 (13.1–20.9)****	25.5 (21.4–30.1)	32.0 (27.4–36.9)	25.9 (22.7–29.3)****
**Age, yrs**				
≤14	23.7 (20.5–27.3)	26.4 (21.8–31.5)	33.9 (30.4–37.7)	16.0 (13.3–19.1)^††††^
15	26.2 (24.1–28.5)^§§§§^	22.8 (21.1–24.7)	32.7 (30.6–35.0)	18.2 (16.2–20.3)
16	23.0 (19.8–26.5)	24.8 (22.6–27.1)	34.1 (30.8–37.5)	18.2 (15.6–21.1)
17	22.5 (19.8–25.3)	24.5 (22.8–26.4)	32.8 (30.1–35.5)	20.3 (17.2–23.7)
**Sexual identity**				
Heterosexual	28.2 (26.3–30.3)^¶¶¶¶,^*****^,†††††,§§§§§^	26.5 (25.2–27.8)*****^,†††††^	32.0 (30.2–33.8)^¶¶¶¶,^*****^,§§§§§^	13.3 (12.1–14.6)^¶¶¶¶,^*****^,†††††,§§§§§^
Gay or lesbian	9.8 (6.4–14.9)	20.3 (14.5–27.8)	44.1 (35.7–53.0)	25.7 (20.7–31.4)^¶¶¶¶¶,^******
Bisexual	9.3 (7.7–11.1)	18.4 (15.8–21.3)^††††††^	37.2 (33.0–41.6)	35.1 (31.7–38.6)^††††††^
Questioning	10.0 (6.6–14.7)	22.9 (19.5–26.6)^§§§§§§^	40.9 (36.0–45.9)	26.3 (21.9–31.3)^§§§§§§^
Describe identity in some other way	9.0 (5.6–14.0)	15.0 (9.6–22.7)	37.3 (29.7–45.6)	38.6 (29.9–48.2)
**Total**	**23.9 (22.0–25.8)**	**24.4 (23.1–25.7)**	**33.3 (31.4–35.2)**	**18.5 (16.5–20.6)**

**Abbreviation:** ACEs = adverse childhood experiences.

* Cumulative ACEs counts were only calculated for participants with complete data on at least five individual ACEs (N = 11,871). The total number of students answering each question varied. Data might be missing because 1) the question did not appear in that student’s questionnaire, 2) the student did not answer the question, or 3) the response was set to missing because of an out-of-range response or logical inconsistency. Percentages in each category are calculated on the known data.

^†^ Male students significantly differed from female students, based on *t*-test analysis with Taylor series linearization (p<0.05).

^§^ Persons of Hispanic or Latino origin might be of any race but are categorized as Hispanic; all racial groups are non-Hispanic.

^¶^ American Indian or Alaska Native students significantly differed from Asian students, based on *t*-test analysis with Taylor series linearization (p<0.05).

** Asian students significantly differed from multiracial students, based on *t*-test analysis with Taylor series linearization (p<0.05).

^††^ Asian students significantly differed from Black or African American students, based on *t*-test analysis with Taylor series linearization (p<0.05).

^§§^ Asian students significantly differed from White students, based on *t*-test analysis with Taylor series linearization (p<0.05).

^¶¶^ Asian students significantly differed from Hispanic or Latino students, based on *t*-test analysis with Taylor series linearization (p<0.05).

*** Black or African American students significantly differed from multiracial students, based on *t*-test analysis with Taylor series linearization (p<0.05).

^†††^ Black or African American students significantly differed from White students, based on *t*-test analysis with Taylor series linearization (p<0.05).

^§§§^ Hispanic or Latino students significantly differed from multiracial students, based on *t*-test analysis with Taylor series linearization (p<0.05).

^¶¶¶^ Hispanic or Latino students significantly differed from White students, based on *t*-test analysis with Taylor series linearization (p<0.05).

**** Multiracial students significantly differed from White students, based on *t*-test analysis with Taylor series linearization (p<0.05).

^††††^ Students aged ≤14 years significantly differed from students aged 17 years, based on *t*-test analysis with Taylor series linearization (p<0.05).

^§§§§^ Students aged 15 years significantly differed from students aged 17 years, based on *t*-test analysis with Taylor series linearization (p<0.05).

^¶¶¶¶^ Heterosexual students significantly differed from gay and lesbian students, based on *t*-test analysis with Taylor series linearization (p<0.05).

***** Heterosexual students significantly differed from bisexual students, based on *t*-test analysis with Taylor series linearization (p<0.05).

^†††††^ Heterosexual students significantly differed from students who describe their identity some other way, based on *t*-test analysis with Taylor series linearization (p<0.05).

^§§§§§^ Heterosexual students significantly differed from students with who were questioning, based on *t*-test analysis with Taylor series linearization (p<0.05).

^¶¶¶¶¶^ Gay or lesbian students significantly differed from bisexual students, based on *t*-test analysis with Taylor series linearization (p<0.05).

****** Gay or lesbian students significantly differed from students who describe their identity some other way, based on *t*-test analysis with Taylor series linearization (p<0.05).

^††††††^ Bisexual students significantly differed from students who were questioning, based on *t*-test analysis with Taylor series linearization (p<0.05).

^§§§§§§^ Students who were questioning significantly differed from students who described their identity some other way, based on *t*-test analysis with Taylor series linearization (p<0.05).

As the number of ACEs increased for most conditions and behaviors, aPRs between cumulative ACEs count and health condition or risk behavior increased in magnitude, indicative of a dose-response relation (Table 4). For 10 of 16 conditions and behaviors, those experiencing one ACE had significantly higher prevalence of each health condition or risk behavior than those experiencing zero ACEs. Students who experienced two or more ACEs had significantly higher prevalence of almost all conditions and behaviors compared with students with zero ACEs (excluding underweight and overweight or obesity for two or three ACEs and underweight for four or more ACEs). The strongest associations were observed between experiencing four or more ACEs and attempted suicide, seriously considered attempting suicide, and current prescription opioid misuse.

**TABLE 4:** Associations between adverse childhood experiences score and health conditions and health risk behaviors among high school students aged <18 years — Youth Risk Behavior Survey, United States, 2023

Outcome^†^	Cumulative adverse childhood experiences*		
	1 (versus 0) Adjusted prevalence ratio^§^ (95% CI)	2 or 3 (versus 0) Adjusted prevalence ratio^§^ (95% CI)	≥4 (versus 0) Adjusted prevalence ratio^§^ (95% CI)
**Violence risk factor**			
Carried a weapon at school	1.57 (0.93–2.64)	2.08 (1.49–2.91)^¶^	4.30 (2.76–6.70)^¶^
Was in a physical fight	1.26 (1.03–1.55)^¶^	2.06 (1.73–2.46)^¶^	3.10 (2.60–3.69)^¶^
**Substance use**			
Current electronic vapor product use	1.72 (1.41–2.10)^¶^	2.92 (2.26–3.78)^¶^	5.26 (4.10–6.76)^¶^
Current alcohol use	1.30 (1.06–1.60)^¶^	1.91 (1.54–2.36)^¶^	2.67 (2.06–3.45)^¶^
Current binge drinking	1.58 (1.13–2.19)^¶^	2.32 (1.74–3.08)^¶^	4.01 (2.80–5.75)^¶^
Current prescription opioid misuse	2.23 (1.32–3.78)^¶^	3.91 (2.18–7.02)^¶^	8.95 (4.98–16.08)^¶^
**Sexual risk behavior**			
Alcohol or drug use before last sexual intercourse	1.90 (1.00–3.64)	2.48 (1.55–3.97)^¶^	7.16 (4.55–11.27)^¶^
Currently sexually active with multiple people	1.19 (0.69–2.08)	1.51 (1.03–2.22)^¶^	3.96 (2.48–6.32)^¶^
Did not use a condom during last sexual intercourse	1.28 (0.94–1.75)	2.03 (1.62–2.54)^¶^	4.03 (2.97–5.47)^¶^
**Weight**			
Underweight	1.27 (0.80–2.01)	0.89 (0.54–1.46)	0.62 (0.37–1.02)
Overweight or obesity	0.91 (0.79–1.06)	1.01 (0.90–1.12)	1.21 (1.07, 1.37)^¶^
Self-perceived to be underweight	1.20 (1.01–1.42)^¶^	1.37 (1.21–1.55)^¶^	1.56 (1.32–1.85)^¶^
Self-perceived to be overweight	1.13 (0.98–1.30)	1.31 (1.13–1.51)^¶^	1.55 (1.37–1.76)^¶^
**Mental health and suicide-related behavior**			
Persistent feelings of sadness or hopelessness	1.94 (1.69–2.22)^¶^	2.75 (2.41–3.14)^¶^	3.81 (3.28–4.42)^¶^
Seriously considered attempting suicide	2.99 (2.17–4.11)^¶^	5.09 (3.71–7.00)^¶^	9.15 (6.86–12.21)^¶^
Attempted suicide	2.20 (1.26–3.84)^¶^	5.22 (3.34–8.17)^¶^	12.42 (7.47–20.65)^¶^

**Abbreviations:** ACEs = adverse childhood experiences; YRBS = Youth Risk Behavior Survey.

* Cumulative ACEs counts were only calculated for participants with complete data on at least five individual ACEs (N = 11,871); the referent group includes those students with zero ACEs. The total number of students answering each question varied. Data might be missing because 1) the question did not appear in that student’s questionnaire, 2) the student did not answer the question, or 3) the response was set to missing because of an out-of-range response or logical inconsistency.

^†^ Because YRBS questionnaires differ by jurisdiction, students are not asked all national YRBS questions. Therefore, the total number of students included in each model varied.

^§^ Adjusted for sex, race and ethnicity, age, and sexual identity.

^¶^ Statistically significant; 95% CIs do not cross the null value of 1.0.

Population-attributable fractions due to ACEs (the potential reduction in each outcome if ACEs were reduced or eliminated in the study population) ranged greatly depending on the outcome and ACEs count (Table 5). The largest potential reductions were estimated among students with four or more ACEs across all health conditions and risk behaviors (range of −6.7% [underweight] to 77.8% [attempted suicide]). The estimated overall potential percentage reductions in negative health conditions and risk behaviors associated with preventing all ACEs (one or more ACE) ranged from 4.2% for overweight or obesity to 89.4% for attempted suicide, with population-attributable fractions exceeding 50% for all conditions and behaviors except those related to weight. Substantial reductions associated with preventing all ACEs were estimated for seriously considered attempting suicide (85.4%), current prescription opioid misuse (84.3%), alcohol or drug use before last sexual intercourse (80.2%), current electronic vapor product use (73.2%), persistent feelings of sadness or hopelessness (65.6%), carrying a weapon at school (65.2%), current binge drinking (64.5%), and not using a condom during last sexual intercourse (64.0%).

**TABLE 5:** Population-attributable fractions for health conditions or risk behaviors among high school students aged <18 years, by cumulative adverse childhood experiences — Youth Risk Behavior Survey, United States, 2023

Health condition or risk behavior	Cumulative adverse childhood experiences*			
	1 ACE Population-attributable fraction,^†^ %	2 or 3 ACEs Population-attributable fraction,^†^ %	≥4 ACEs Population-attributable fraction,^†^ %	Any ACE (≥1 ACE)
**Weapon carrying and violence**				
Carried a weapon at school	3.3	8.8	53.1	65.2
Was in a physical fight	2.2	13.9	37.3	53.4
**Substance use**				
Current electronic vapor product use	3.1	13.5	56.6	73.2
Current alcohol use	2.9	12.4	33.8	49.2
Current binge drinking	3.6	11.9	48.9	64.5
Current prescription opioid misuse	2.7	10.6	71.0	84.3
**Sexual behavior**				
Alcohol or drug use before last sexual intercourse	2.6	5.6	72.0	80.2
Currently sexually active with multiple persons	1.3	3.9	56.1	61.3
Did not use a condom during last sexual intercourse	1.6	8.9	53.5	64.0
**Weight**				
Underweight	9.1	−2.6	−6.7	−0.2
Overweight or obesity	−1.8	0.2	5.8	4.2
Self-perceived to be underweight	3.4	7.3	13.7	24.3
Self-perceived to be overweight	2.3	6.3	13.7	22.3
**Mental health and suicide-related behavior**				
Persistent feelings of sadness or hopelessness	6.1	17.7	41.8	65.6
Seriously considered attempting suicide	4.2	16.1	65.1	85.4
Attempted suicide	1.1	10.6	77.8	89.4

**Abbreviation:** ACEs = adverse childhood experiences.

* Cumulative ACEs counts were only calculated for participants with complete data for at least five individual ACEs (N = 11,871); the referent group includes those students with zero ACEs. The total number of students answering each question varied. Data might be missing because 1) the question did not appear in that student’s questionnaire, 2) the student did not answer the question, or 3) the response was set to missing because of an out-of-range response or logical inconsistency.

^†^ Population-attributable fraction calculated using Miettinen’s formula and weighted after adjusting for sex, race and ethnicity, age, and sexual identity.

## Discussion

This study is the first to present self-reported, nationally representative estimates of ACEs among U.S. high school students. ACEs were common among students, exceeding previous estimates from U.S. adults (*4*): Three in four students experienced one or more ACE and nearly one in five students experienced four or more ACEs. Approximately 60% of students experienced emotional abuse during their lifetime (61.5%) and approximately one in three (31.8%) experienced physical abuse. More than one in four students live in a household that has been affected by substance use (25.1%) or poor mental health (28.4%). Approximately one in five students has witnessed intimate partner violence (18.6%), and one in seven has had a parent or guardian incarcerated or detained (14.5%). Students who identified as female, AI/AN, multiracial, or LGBQ+ experienced the highest prevalence of ACEs. Results indicate that preventing and mitigating ACEs is critical to improving population-level adolescent behavioral health: Nearly 90% of suicidal behaviors, 84.3% of current prescription opioid misuse, and 65.6% of persistent feelings of sadness or hopelessness were associated with experiencing one or more ACE. Students with four or more ACEs had significantly increased prevalence ratios for 15 of 16 negative health conditions and risk behaviors compared with students with zero ACEs (e.g., aPR for attempted suicide = 12.42), demonstrating the marked association between cumulative ACEs and negative outcomes.

Results highlight the wide variety of individual student experiences across and within racial and ethnic groups. For example, although AI/AN students had the highest prevalence of four or more ACEs, they also had the highest prevalence of zero ACEs. Specific ACEs most prevalent among students differed by racial and ethnic identity, consistent with previous studies (*12*). For example, although Asian students had the lowest prevalence of four or more ACEs, they experienced comparatively high prevalence of emotional and physical abuse. Significantly fewer multiracial students experienced zero ACEs compared with all other racial and ethnic groups, except when compared to AI/AN students. Taken together, these findings indicate important racial and ethnic disparities in exposure to individual and cumulative ACEs. Variability of individual ACEs by racial and ethnic identity indicates that approaches designed to prevent or address the impact of ACEs might benefit from being tailored to specific cultural contexts, as opposed to a one-size-fits-all approach.

Although LGBQ+ students experienced a higher prevalence of ACEs than heterosexual students, bisexual students also experienced a disproportionate prevalence of certain ACEs compared with gay and lesbian students. These findings align with previous research, though the reason for the disproportionate prevalence of ACEs among bisexual students compared with gay and lesbian students remains unclear and needs to be further explored to guide prevention efforts (*13*). Students who described their sexual identity in some other way demonstrated the highest prevalence of four or more ACEs, significantly higher than heterosexual, gay and lesbian, and questioning students; these findings support the need to better understand this population of students and their prevention and intervention needs.

Female students had significantly higher prevalence of four or more ACEs than male students and had higher prevalence of all individual types of ACEs except for physical neglect. Although certain ACEs (e.g., physical and sexual abuse) might reflect directed violence based on their sex, other ACEs (e.g., household poor mental health) might instead reflect a greater awareness of household challenges among female students compared with male students.

Although it might be expected that older students would have experienced a greater number of ACEs than younger students, this study found only a modest increase between students aged ≤14 years and those aged 17 years. The only two ACEs that significantly increased between those aged ≤14 years and those aged 17 years were household substance use and household poor mental health, which more likely reflects greater awareness of household challenges among older adolescents than a true increase in prevalence. This supports the approach that preventing ACEs earlier in childhood helps to prevent negative impacts in adolescence and beyond.

Results from this study expand upon previous research indicating that preventing ACEs could result in sizable reductions in poor health conditions and risk behaviors among adult populations (*7*). However, this study is the first to document the critical, widespread contribution that preventing ACEs could have on reducing a wide spectrum of poor behavioral health conditions and risk behaviors among adolescents. With a high association of ACEs with attempted suicide (89.4%), with current prescription opioid misuse (84.3%), and with persistent feelings of sadness or hopelessness (65.6%), preventing ACEs could be a clear pathway to improving adolescent behavioral health amid an ongoing mental health crisis (*5*).

Students experiencing high cumulative (four or more) ACEs had significantly higher prevalence of overweight or obesity, consistent with previous studies linking ACEs with obesity (*2*). Such findings suggest the need to incorporate ACEs prevention and mitigation into interventions and policies that aim to address childhood obesity, such as incorporating social workers into family healthy weight programs to assist families in accessing benefit programs (e.g., Supplemental Nutrition Assistance Program or Women, Infants, and Children) that might alleviate financial stress and promote food security, and integration of Substance Abuse and Mental Health Services Administration’s guiding principles for trauma-informed care into behavioral interventions for weight (*14*).

Preventing ACEs is important because all children deserve to have safe, stable, nurturing relationships and environments in childhood so that they can flourish and thrive; experiencing abuse and neglect and witnessing violence disrupt that safety, stability, and nurturing. However, these findings suggest that preventing ACEs also is an important public health goal because many other health problems could be eliminated or reduced when ACEs are prevented (*7*). Preventing ACEs is possible and achievable, and CDC’s Preventing Adverse Childhood Experiences Resource for Action outlines six strategies for preventing ACEs and mitigating their negative consequences based on the best available evidence (*1*). This resource outlines approaches for advancing each strategy, with examples of evidence-based programs, policies, and practices for each approach. Strategies include increasing economic supports for families, promoting social norms that protect against ACEs, ensuring a strong start for children, teaching skills, and connecting youth to caring adults and activities. Tailored approaches to address the most prevalent ACEs experienced by U.S. high school students are needed. Examples include promoting of parenting skills and family relationship approaches to prevent emotional abuse, providing high-quality childcare to reduce risk for physical abuse and improve household mental health, and offering family-centered treatment for substance use disorders (*1*). Because more than three fourths of U.S. high school students have experienced ACEs, intervening to lessen long-term harms of ACEs is critical for improving public health. Policymakers and public health professionals in states and communities that implement evidence-based approaches to advance these strategies could prevent ACEs among children, and this study’s findings suggest that preventing ACEs might also translate into sizable reductions in suicidal behaviors, substance use, sexual risk behaviors, violence, and persistent feelings of sadness and hopelessness when these children reach adolescence.

## Limitations

General limitations for the YRBS are available in the overview report of this supplement (*8*). The findings in this report are subject to at least four additional limitations. First, although the eight ACEs categories measured on the YRBS are consistent with traditional measures of ACEs (*3*), these categories do not capture all potentially traumatic experiences in childhood or characteristics such as intensity or severity of the experience, which might have implications for health conditions and risk behaviors. This study dichotomized ACEs exposures; future studies could use available responses to explore the nuances of ACEs frequency on conditions and behaviors. Second, these data are cross-sectional. Whereas the ACEs questions measure lifetime occurrence and most conditions and behaviors of interest reflect current (past 30 days) or past-year experiences, for certain persons, ACEs might not have occurred before the outcome of interest. These effects, although strong and consistent, are correlative in nature and should not be interpreted as causal associations between ACEs and included risk behaviors and health conditions. Third, social determinants of health and other related risk factors that contribute to both ACEs and health conditions and risk behaviors could not be assessed, but these social and structural conditions shape the contexts in which adolescents live, play, and learn. Not analyzing these factors could lead to inaccurate estimations of the relations between ACEs and conditions and behaviors because the survey did not include underlying factors with strong influence on the context that shapes adolescents’ lives and communities. Finally, adolescents who have experienced multiple ACEs might be more likely to be engaged with the juvenile justice system, unstably housed, or to miss school frequently (*15*), all of which could affect their opportunity to participate in the school-based YRBS. As a result, estimates for ACEs might be underreported.

## Future Directions

The findings in this report highlight the importance of preventing ACEs for adolescent well-being and as a prevention and reduction strategy for multiple other adolescent health and behavioral risks, such as suicide risk, substance use, sexual risk behaviors, and poor mental health. Ongoing monitoring of ACEs among adolescents is critical to understand trends in the prevalence of ACEs, inequities in the number of ACEs, and effects of ACEs prevention efforts over time. Future research in this area might examine how individual ACEs uniquely contribute to health conditions and risk behaviors, including across demographic groups, and identify protective factors and positive childhood experiences that might buffer against the negative effect of ACEs on adolescent health conditions and risk behaviors. In 2023, CDC’s National Center for Injury Prevention and Control partnered with the Division of Adolescent and School Health to obtain national level estimates of ACEs. Ongoing research and surveillance are warranted to support data-driven, evidence-based implementation of primary prevention approaches to prevent ACEs and to mitigate their harms. CDC supports a robust portfolio across the public health model to better monitor, understand, prevent, and respond to ACEs so that all children and families can thrive (*1*).

## Conclusion

ACEs are common in U.S. high school students and have significant associations with negative health conditions and risk behaviors. Prevalence of individual ACEs differ by demographic characteristics, with the highest prevalence of ACEs disproportionately affecting female, AI/AN, multiracial, and LGBQ+ students. Preventing ACEs has considerable potential public health impact in adolescence and beyond, with substantial population-attributable fractions (>50%) for all violence, substance use, sexual, mental health, and suicide-related behaviors. Timely, adolescent-reported data on ACEs is needed to tailor prevention strategies to specific cultural contexts and populations. CDC’s Preventing Adverse Childhood Experiences Resource for Action provides evidence-based strategies and approaches to prevent ACEs and mitigate their consequences (*1*).

## Footnotes

* 45 C.F.R. part 46.114; 21 C.F.R. part 56.114.
